# Numerical Study on Characteristics of Convection and Temperature Evolution in Microchannel of Thermal Flowmeter

**DOI:** 10.3390/mi14050935

**Published:** 2023-04-25

**Authors:** Hang Che, Qingxuan Xu, Guofeng Xu, Xinju Fu, Xudong Wang, Naifeng He, Zhiqiang Zhu

**Affiliations:** 1 Institute of Mechanics, Chinese Academy of Sciences, Beijing 100190, China; chehang22@mails.ucas.ac.cn (H.C.);; 2 University of Chinese Academy of Sciences, Beijing 100049, China; 3 Beijing Institute of Control Engineering, Beijing 100190, China; 4 Beijing Engineering Research Center of Efficient and Green Aerospace Propulsion Technology, Beijing 100190, China

**Keywords:** numerical simulation, thermal flowmeter, forced convection, buoyancy convection, microchannel heat source

## Abstract

During practical usage, thermal flowmeters have a limited range of applications. The present work investigates the factors influencing thermal flowmeter measurements and observes the effects of buoyancy convection and forced convection on the flow rate measurement sensitivity. The results show that the gravity level, inclination angle, channel height, mass flow rate, and heating power affect the flow rate measurements by influencing the flow pattern and the temperature distribution. Gravity determines the generation of convective cells, while the inclination angle affects the location of the convective cells. Channel height affects the flow pattern and temperature distribution. Higher sensitivity can be achieved with smaller mass flow rates or higher heating power. According to the combined influence of the aforementioned parameters, the present work investigates the flow transition based on the Reynolds number and the Grashof number. When the Reynolds number is below the critical value corresponding to the Grashof number, convective cells emerge and affect the accuracy of flowmeter measurements. The research on influencing factors and flow transition presented in this paper has potential implications for the design and manufacture of thermal flowmeters under different working conditions.

## 1. Introduction

Thermal flowmeters, specifically calorimetric flowmeters, are widely used in a variety of industrial manufacturing, aerospace, biomedical engineering, and other fields. Because of their compact size, quick response time, and low energy consumption, they are particularly useful for microflow rate measurements [1,2]. The thermal flowmeter works as follows: when the working medium flows over the upstream and downstream heat sources inside the thermal flowmeter channel successively, heat is carried by the flow simultaneously, resulting in a temperature difference between the upstream and downstream heat sources. The flow rate evolution can be obtained by accurately measuring the temperature difference after calibration [3].

Despite their advantages, thermal flowmeters have certain limitations. For example, they tend to saturate at high flow rates, thus affecting the measuring accuracy. Additionally, the heat loss increases power consumption and reduces device sensitivity [4]. Furthermore, factors such as the distribution of heat sources in the flow channel, the wall material, the size of the flow channel, the mass flow rate, the power of the heat source, and the gravity level of the environment where the thermal flowmeter is located may all impact the measurement accuracy [5,6].

In the calculation of thermal flowmeter flow and heat transfer, the device structure is commonly simplified as a one-dimensional channel with a single source [7], two-dimensional channel with single [8,9,10,11,12] or multiple [13,14,15,16] heat sources, and three-dimensional channel with multiple heat sources [17]. Komiya [7] was the first to provide an analytical solution for the one-dimensional thermal flowmeter. Two-dimensional models with a single heat source typically only consider the intermediate heat source, while ignoring the power of the upstream and downstream sources. Studies have shown that the maximum temperature of the heat source decreases as the thermal conductivity of the channel wall material increases [9], and that the position of the heat source in the vertical channel has a negligible effect on the flow [10]. Choi et al. [11] found that the maximum surface temperature in the flow channel does not always decrease with increasing flow in the horizontal case. Studies with multiple heat sources have found that increasing the heat source spacing increases the flow velocity near the heat sources and reduces the maximum temperature [13]. When the heat source distance exceeds three times of the heat source size, the heat transfer between the two heat sources has no effect [14]. However, the positions of the upstream and downstream sources have an impact on the sensitivity of the flow rate measurement. When the distance of the temperature measuring sources is closer, the measurement sensitivity is higher [18]. Cheng et al. [17] established a three-dimensional channel model with four heat sources and found that the channel height and the inclination angle have significant influences on the overall Nusselt number when the heat sources are at constant temperatures. In addition, many scholars have studied the impact of other factors on heat transfer, such as the wall structure [19,20,21,22] and the working fluid [23].

Recently, with the development of space exploration, accurate flow rate measurement has been found to play a critical role and have potentially important applications in many space industries, such as power control of micro/nano satellite propulsion systems and fluid transport control of space life support systems. Nevertheless, in the normal gravity environment on the ground, the existence of buoyancy convection significantly affects flow and thermal characteristics, resulting in discrepancies between ground and space flow rate measurements. Furthermore, calibrated equipment is prone to errors in space [6,24,25], making it necessary to analyze the influence of gravity level on the flowmeter. The numerical model demonstrated that the buoyancy-driven recirculating eddies induced by heated sensors led to lower accuracy of hot-film MEMS thermal flow sensors [26]. When the flow velocity increases to a certain value, the influence of buoyancy convection on the flow can be ignored [27,28]. Some studies found that changing the angle between the flow channel and gravity causes errors in the measurements [5,29]. However, the survey found that previous studies did not focus on the effect of gravity on buoyancy convection in a channel, which is critical for accurate flow rate measurements. Therefore, in simulations of thermal flowmeters with low flow rates, the effect of gravity is considered to study applicability in accurate flow rate measurement in the present work.

In addition to the influence of gravity level, the accuracy of flowmeter measurement is also related to the temperature difference between upstream and downstream heat sources. When the working fluid flows through the channel, heat is transferred from the channel to the fluid on the upstream source, and from the fluid back to the downstream source, creating a temperature difference between the upstream and downstream heat sensors. The temperature difference is eventually converted into a measurement of the mass flow rate. It is noted that previous studies mainly focused on the change of flow and temperature fields. On the other hand, the present work performs relatively comprehensive studies to focus on the influence of the inclination angle, channel height, mass flow rate, and heating power on the temperature difference between upstream and downstream heat sources from the perspective of the flowmeter mechanism.

This paper presents a numerical investigation of liquid in a two-dimensional rectangular thermal flowmeter channel, which is subjected to three equally spaced constant heat sources. The finite element method is employed to solve the governing equations for the model. Typical dimensionless parameters are derived to normalize the governing equations and to define the dimensionless boundary conditions.

A grid independence verification and validation of the model are carried out, and the effects of gravity level, inclination angle, flow channel height, mass flow rate, and heating power on the flow field, temperature field, temperature at the heat sources, and the temperature difference between the upstream and downstream heat sources are analyzed. Lastly, the effects are synthesized into the Grashof number and the Reynolds number, to investigate their influence on the transition of flow patterns.

The analysis presented in this paper provides novel insights into the influence of buoyancy and forced convection in thermal flowmeters. The findings of this study have potential implications for the design and optimization of thermal flowmeters used in various industrial applications.

## 2. Problem Formulation

### 2.1. Problem Description

**Figure 1:**
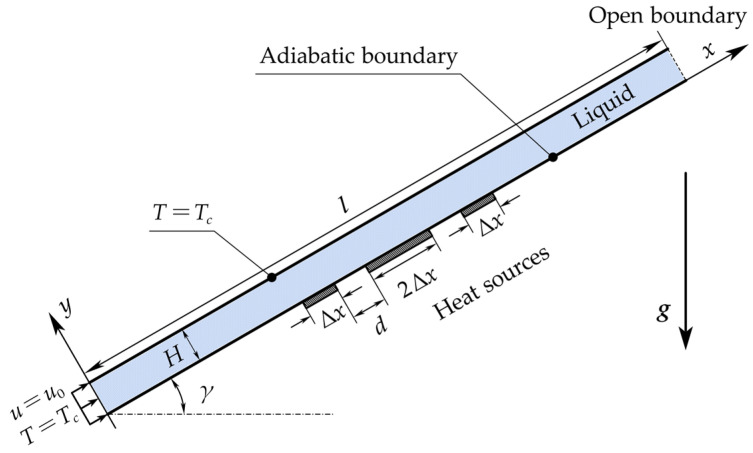
Schematic of the mathematical model. The blue region represents liquid.

The physical model is depicted in Figure 1, illustrating the steady combined forced and buoyancy convection of liquid within a two-dimensional rectangular thermal flowmeter channel, which is subjected to three equally spaced constant heat sources. The channel length (*l*), height (*H*), and depth (*d_z_*), along with the distance between heat sources (*d*), length of the intermediate heat source (2*Δx*), and length of the upstream and downstream heat sources (*Δx*), are presented. The inclination angle of the channel (*γ*) refers to the angle between the channel axis and the horizontal direction.

The temperature of the wall opposite to the heat sources is *T_c_*, which is constant. The wall where the heat sources located is adiabatic. The direction of the gravity is vertically downward. The inlet velocity (*u*_0_) is also considered. The heating power of the intermediate heat source (*P*/2) and the upstream and downstream heat sources (*P*/4), along with the total heating power of the three heat sources (*P*), is also described.

There are numerous working fluids that can be utilized in liquid thermal flowmeters. This study employs the commonly used deionized water as the working fluid. The physical properties of the deionized water are presented in Table 1.

### 2.2. Governing Equations

The flow in the channel is assumed to be incompressible, two-dimensional, and in a steady state. Considering that the flow channel length is long enough, it is assumed that the flow has reached the fully developed section. The Boussinesq approximation is used to describe the buoyancy effect. The thermophysical properties in the liquid are assumed to be constant at the reference temperature *T* = *T_c_* except for the fluid density *ρ* in the buoyancy terms of the Navier–Stokes equations, in which it varies linearly with the local temperature as *ρ* = *ρ*_0_ [*1* − *β* (*T − T_c_*)], where *β* is the coefficient of thermal expansion. The following dimensional governing equations hold:

(1) ∇⋅u=0,

(2) $$\frac{\partial u}{\partial t}+u\cdot \nabla u=-\frac{1}{\rho_{0}}\nabla p+\nu \nabla^{2}u+g[1-\beta (T-T_{c})],$$

(3) $$\frac{\partial T}{\partial t}+u\cdot \nabla T=a\cdot \nabla^{2}T.$$

In the dimensional model, *l* = 50 mm, *d_z_* = 1 mm, *d* = 1 mm, and *Δx =* 1 mm. We use *u*_0_, *H*_0_, *ρ*_0_*u*_0_^2^, and *qH*_0_/*k* as scales for velocity, length, pressure, and temperature, respectively. *U*_0_, *H*_0_, *ρ*_0_, and *q* are the inlet velocity, channel height, reference density, and heat flux, respectively [31,32]. The dimensionless variables are defined as follows:

$$\bar{u}=\frac{u}{u_{0}},\;\bar{l}=\frac{l}{H_{0}},\;\bar{H}=\frac{H}{H_{0}},\;\bar{t}=\frac{t}{H_{0}/u_{0}},\;\bar{p}=\frac{p}{\rho_{0}u_{0}{}^{2}},\;\theta =\frac{T-T_{c}}{qH_{0}/k},$$

where $\bar{u}$, $\bar{l}$, $\bar{H}$, $\bar{t}$, $\bar{p}$, and *θ* represent the dimensionless velocity vector, length of the channel, height of the channel, time, pressure, and temperature, respectively. $Pr=\frac{\nu }{a}$ is the Prandtl number, where $a=\frac{k}{\rho_{0}C_{p}}$ is the thermal diffusivity. $Re=\frac{\rho_{0}u_{0}H}{\mu }=\frac{Q}{\mu d_{z}}$ is the Reynolds number indicating the strength of forced convection, where *Q* = *ρ*_0_*u*_0_ *Hd_z_* is the mass flow rate. $Gr=\frac{g\beta q\rho_{0}{}^{2}H^{4}}{\mu^{2}k}$ is the Grashof number indicating the strength of buoyancy convection. The following dimensionless governing equations hold:

(4) $$\nabla \cdot \bar{u}=0,$$

(5) $$\frac{\partial \bar{u}}{\partial \bar{t}}+\bar{u}\cdot \nabla \bar{u}=-\nabla \bar{p}+\frac{1}{Re}\nabla^{2}\bar{u}+\theta \frac{Gr}{Re^{2}}i,$$

(6) $$\frac{\partial \theta }{\partial \bar{t}}+\bar{u}\cdot \nabla \theta =\frac{1}{Re\cdot Pr}\cdot \nabla^{2}\theta ,$$

where ***i*** = (sin *γ,* cos *γ*).

### 2.3. Boundary Conditions

Since the constant temperature wall is usually made of materials with better thermal conductivity, it is assumed that the constant wall temperature *T* is the same as ambient temperature *T_c_*. In order to reduce the heat loss of the wall, the boundary of the wall where the heat sources are located is set as adiabatic except for the heat sources, the outlet is an open boundary condition, and the wall has no slip. The initial and boundary conditions are as follows:

$$\bar{U}=1,\;\bar{V}=0;\;\theta =0,\;\mathrm{at}\;\bar{x}=0,$$

$$\frac{\partial \bar{U}}{\partial \bar{x}}=0,\;\bar{V}=0;\;\frac{\partial \theta }{\partial \bar{x}}=0,\;\mathrm{at}\;\bar{x}=\bar{l},$$

$$\bar{U}=0,\;\bar{V}=0;\;\theta =0,\;\mathrm{at}\;\bar{y}=1,$$

$$\bar{U}=0,\;\bar{V}=0;\;\frac{\partial \theta }{\partial \bar{y}}=0,\;\mathrm{outside}\;\mathrm{the}\;\mathrm{heat}\;\mathrm{sources},\;\mathrm{at}\;\bar{y}=0,$$

$$\bar{U}=0,\;\bar{V}=0;\;\frac{\partial \theta }{\partial \bar{y}}=-1,\;\mathrm{at}\;\mathrm{heat}\;\mathrm{sources},$$

where $\bar{x}$ *= x*/*H*_0_ and $\bar{y}$ *= y*/*H*_0_ are dimensionless streamwise coordinate and dimensionless transverse coordinate, respectively. $\bar{U}$ is the dimensionless velocity component in the *x*-direction, and $\bar{V}$ is the dimensionless velocity component in the *y*-direction.

### 2.4. Numerical Method

The commercial software COMSOL Multiphysics is used to solve this coupled system of fluid flow and heat transfer. The governing equations of the flow field and the temperature field along with the boundary conditions are solved using the finite element method. The P2 + P2 (velocities and pressures are both approximated using a second-order equation), and steady-state fully coupled solution methods are adopted. Furthermore, the PARDISO solver accelerates the calculation speed. The maximum relative tolerance on the dependent variable (*u*, *p*, and *T*) was lower than 0.1% during the iterative process [33].

## 3. Grid Independence Study

The grid dependency is checked for four stretched and structured grids *N_x_* × *N_y_*, where *N_x_* and *N_y_* are the numbers of grids in the *x*- and *y*-directions, respectively. Typical results at *Q* = 855 μg/s, *P* = 100 mW, *H* = 1 mm, and *γ* = 0° are listed in Table 2. In general, the difference between 460 × 40 and 530 × 50 mesh is less than 0.03%, while the calculation time increased by 46%. Thus, the 460 × 40 mesh was selected.

## 4. Model Validation

**Figure 2:**
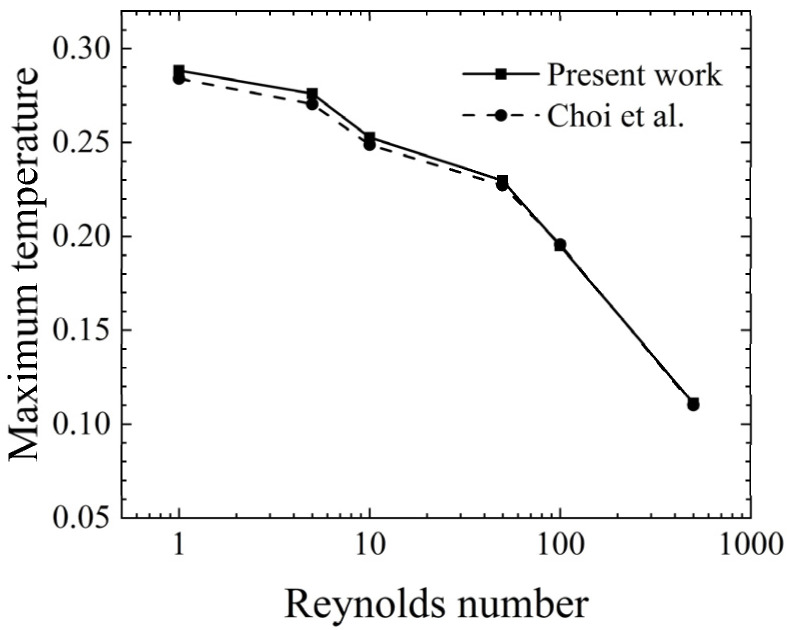
Validation of the present work and the Choi et al. model. Data from [11].

In order to validate the numerical model proposed in this study, simulations were conducted for a channel with a single heat source. Specifically, the dimensionless temperature on the lower wall was obtained for a horizontally positioned channel and compared with the simulation results of the classical numerical model developed by Choi et al. [11]. The results are presented in Figure 2.

It was observed that the error between the current numerical model and the work of Choi et al. [11] was 1.5% when *Re* = 1. However, as the Reynolds number increased, the error gradually decreased. This finding suggests that the proposed numerical model is in excellent agreement with the classical numerical model developed by Choi et al. [11], which further validates the numerical simulation results presented in this paper.

**Figure 3:**
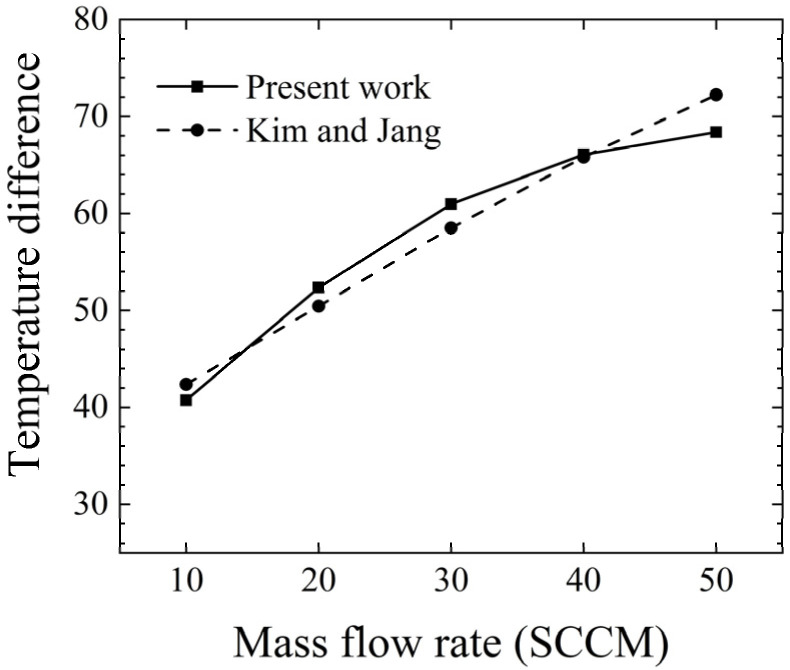
Validation of the present work and the experimental result of Kim and Jang. Data from [12].

Furthermore, the comparison with the experimental result of Kim and Jang [12], which obtained the temperature difference between the temperature downstream of the flow sensor and the surrounding temperature, is given in Figure 3. In the measured flow range, the relative error between the present work and Kim and Jang’s [12] experimental results was less than 5.36%. The correctness of the computation is verified.

## 5. Results and Discussion

In the present paper, the influence of buoyancy convection on the liquid flow and heat transfer within a thermal flowmeter was studied under different conditions, including the gravity level, the inclination angle and height of the flow channel under gravity, mass flow rate, and heating power. The highest temperature considered in the calculations did not exceed the boiling point of water at standard atmospheric pressure; therefore, phase change was not considered.

### 5.1. Gravity Level

**Figure 4:**
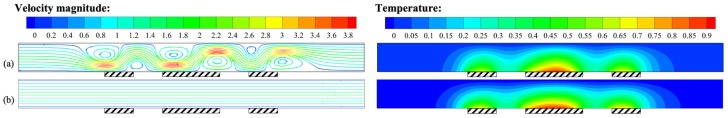
Velocity field and temperature field for *Q* = 85.5 μg/s, *H* = 1 mm, *γ* = 0°, and *P* = 100 mW: (**a**) normal gravity; (**b**) zero gravity.

The flow field and temperature distribution of the liquid inside the channel were calculated under the presence and absence of gravity, as shown in Figure 4. When gravity was present, the streamlines became curved, and the isotherms near the heat sources protruded upward along the flow direction. The liquid in the pipeline was subjected to both buoyancy convection and forced convection. In the absence of gravity, the streamlines were straighter, and the temperature distribution near the heat sources was more symmetric. In this case, the fluid was only affected by forced convection. The above results indicate that gravity played a decisive role in the transition of flow patterns.

**Figure 5:**
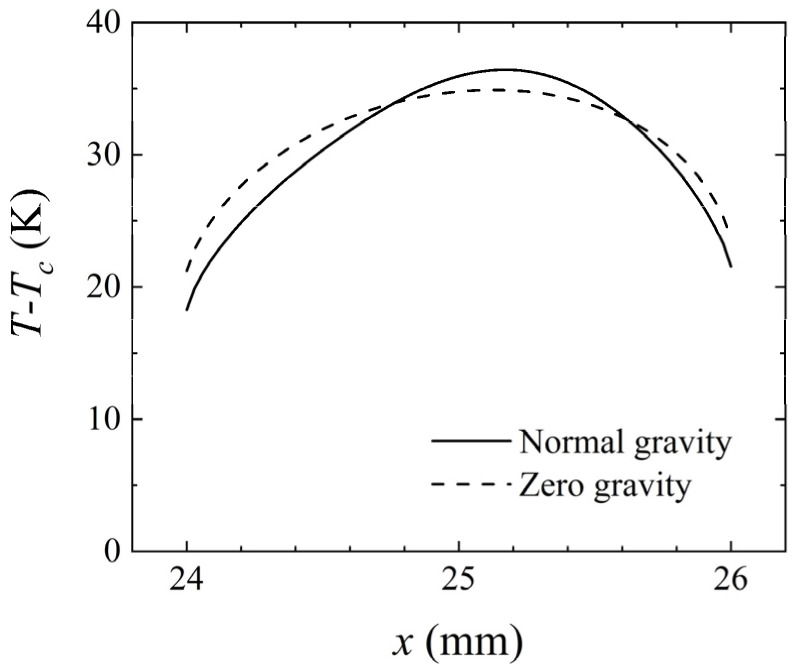
Variation of *T*–*T_c_* at the intermediate heat source (24–26 mm) for *Q* = 85.5 μg/s, *H* = 1 mm, *γ* = 0°, and *P* = 100 mW at different gravity levels.

To evaluate the impact of buoyancy convection on the temperature of the intermediate heat source, Figure 5 is presented. It can be observed that the steady-state convective cells led to a more concentrated temperature distribution at the intermediate heat source. In the presence of gravity, the convective cells disturbed the original forced convection flow field, thereby affecting the temperature distribution and the temperature difference between the upstream and downstream heat sources. Therefore, in a gravitational environment, the maximum temperature at the central heat source was higher, and the temperature difference between the upstream and downstream heat sources increased from 1.75 K to 1.86 K, an increase of 6.72% in temperature difference resulting in a measurement error of mass flow rate toward higher values.

### 5.2. Inclination Angle of the Channel

**Figure 6:**
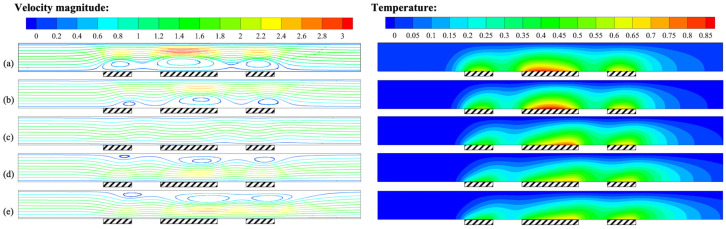
Velocity field and temperature field for *Q* = 427.5 μg/s, *H* = 1 mm, and *P* = 100 mW: (**a**) *γ* = −90°; (**b**) *γ* = −45°; (**c**) *γ* = 0°; (**d**) *γ* = 45°; (**e**) *γ* = 90°.

In practical applications of thermal flowmeters, the flow channel may not be horizontal due to improper installation, which can adversely affect the accurate measurement of mass flow rate. To quantify the effect of the inclination angle on the flow field and temperature, numerical simulations were performed for different inclination angles, as shown in Figure 6.

At an inclination angle of 0°, i.e., when the channel was horizontal, the strength of buoyancy convection and forced convection was comparable, and the streamlines exhibited slight curvature. The flow pattern was in a steady state in this condition. At an inclination angle less than 0°, i.e., when the channel inlet was located above the outlet, buoyancy convection caused the velocity near the center of the constant temperature wall to increase compared to the inlet velocity, and the direction of the velocity near the heat sources reversed, as the convective cells were close to the heat sources. The isotherm at the heat sources was inclined upstream due to buoyancy convection. In this condition, buoyancy convection and forced convection were in opposition. For an inclination angle greater than 0°, i.e., when the channel inlet was located below the outlet, buoyancy convection caused the velocity near the heat sources to increase, the direction of the velocity near the constant temperature wall to reverse, and the isotherm at the heat sources to be inclined downstream, as the convective cells were close to the isothermal wall. In this condition, buoyancy convection and forced convection were in synergy.

**Figure 7:**
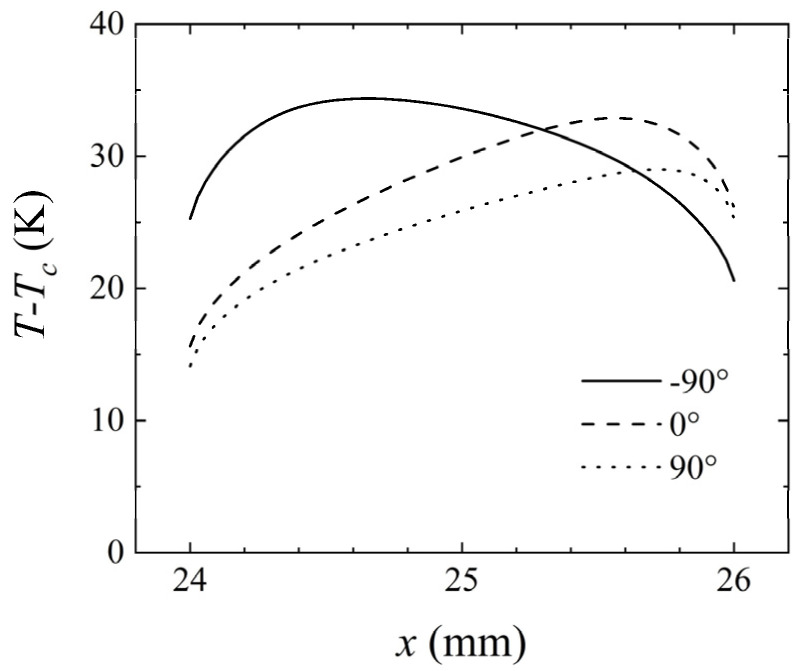
Variation of *T*–*T_c_* at the intermediate heat source (24–26 mm) for *Q* = 427.5 μg/s, *H* = 1 mm, and *P* = 100 mW at different inclination angles of the channel.

**Figure 8:**
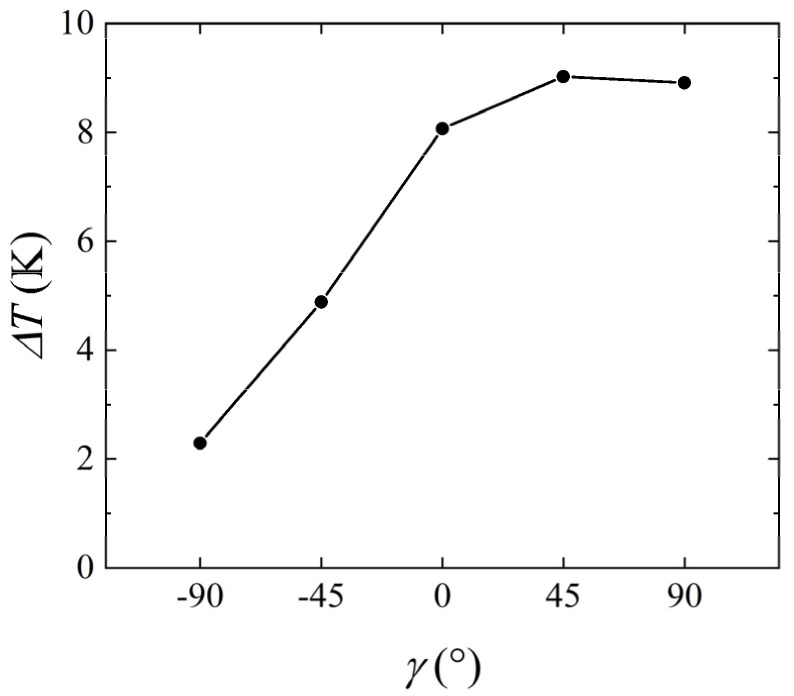
Variation of *ΔT* with the inclination angle of the channel for *Q* = 427.5 μg/s, *H* = 1 mm, and *P* = 100 mW.

As mentioned above, different flow patterns usually lead to varying temperature differences. Figure 7 was calculated to describe the temperature at the intermediate heat source under different inclination angles. Using the channel placed horizontally as a reference, when *γ* = −90°, the maximum temperature at the intermediate heat source increased, and the upstream temperature of the intermediate heat source was higher than that downstream. When *γ* = 90°, the maximum temperature at the intermediate heat source decreased, while the downstream temperature of the intermediate heat source was higher than that upstream. Figure 8 shows the change in temperature difference between the upstream and downstream heat sources with the inclination angle. Using *γ* = 0° as a reference, the temperature difference between the upstream and downstream heat sources was 8.07 K. When *γ* = −90°, the temperature difference was 2.29 K, and this value was 8.91 K for *γ* = 90°. The above results indicate that the inclination angle of the channel also had a significant impact on the measurement of thermal flowmeters. Therefore, to ensure measurement accuracy, the inclination angle of the thermal flowmeter must be consistent during calibration and measurement.

### 5.3. Height of the Channel

**Figure 9:**
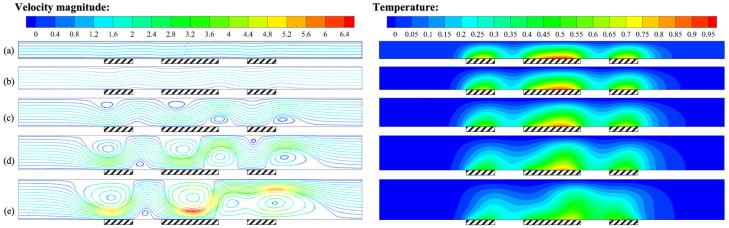
Velocity field and temperature field for *Q* = 171 μg/s, *γ* = 0°, and *P* = 100 mW: (**a**) *H* = 0.6 mm; (**b**) *H* = 0.8 mm; (**c**) *H* = 1.0 mm; (**d**) *H* = 1.2 mm; (**e**) *H* = 1.4 mm.

In Figure 9, the influence of the channel height on the flow field and temperature is shown. When *H* < 0.8 mm, the streamlines were straight, with forced convection being more significant than buoyancy convection. After *H* > 0.8 mm, the streamlines became tortuous, the isotherm near the heat sources bulged upward along the flow direction, showing the effect of buoyancy convection, and the flow pattern transformed to steady unicellular flow. As *H* continued to increase, the degree of streamlines curvature increased, and the isotherm near the heat sources moved to the upper right, with the effect of buoyancy convection gradually becoming dominant.

**Figure 10:**
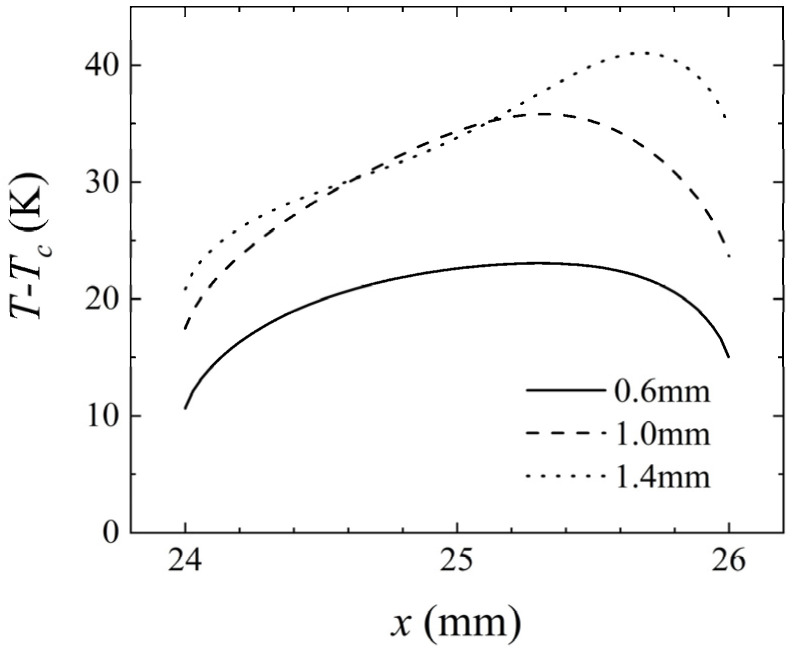
Variation of *T*–*T_c_* at the intermediate heat source (24–26 mm) for *Q* = 171 μg/s, *γ* = 0°, and *P* = 100 mW at different heights of the channel.

Figure 10 shows the variation of temperature at the heat sources in the channel at different heights. As the height of the channel increased, the distance between the upper cold wall and the heat sources increased, thereby reducing its influence and causing the heating temperature to increase.

Specifically, when *H* = 1.4 mm, the position of the highest temperature in the heat sources moved to the right. The distance between the heat sources was relatively small compared to the height of the flow channel, resulting in a mutual influence of the buoyancy convective cells generated by adjacent heat sources. This also caused abnormally high temperatures downstream of the intermediate heat source.

**Figure 11:**
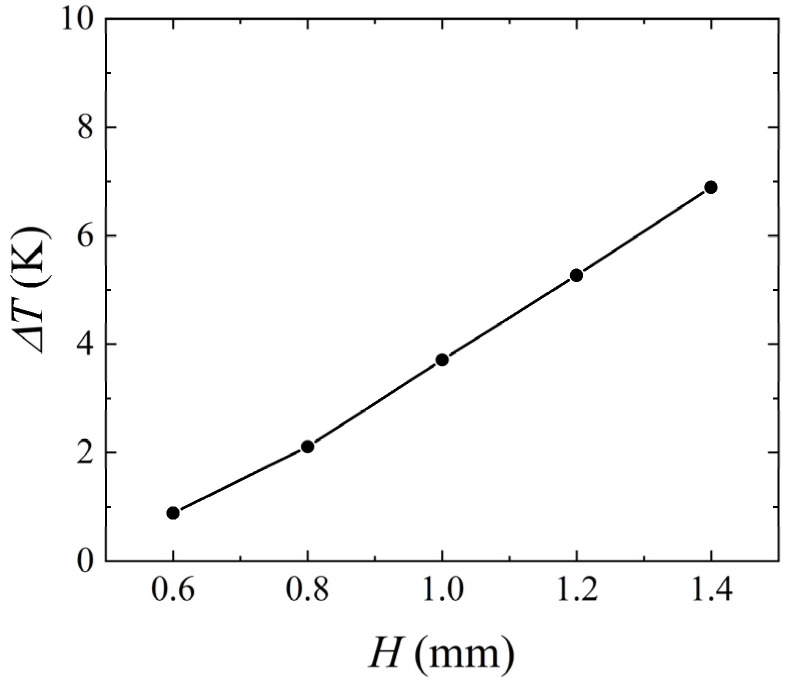
Variation of *ΔT* with the height of the channel for *Q* = 171 μg/s, *γ* = 0°, and *P* = 100 mW.

In order to further investigate the effect of height on the measurement accuracy of the thermal flowmeter, Figure 11 describes the variation of the temperature difference between the upstream and downstream heat sources with respect to *H*. As *H* increased from 0.6 mm to 1.4 mm, buoyancy convection gradually became dominant, causing the temperature difference between the upstream and downstream heat sources to increase from 0.89 K to 6.89 K. Overall, the analysis above indicated that the channel height significantly affected the temperature difference between upstream and downstream heat sources.

### 5.4. Mass Flow Rate

**Figure 12:**
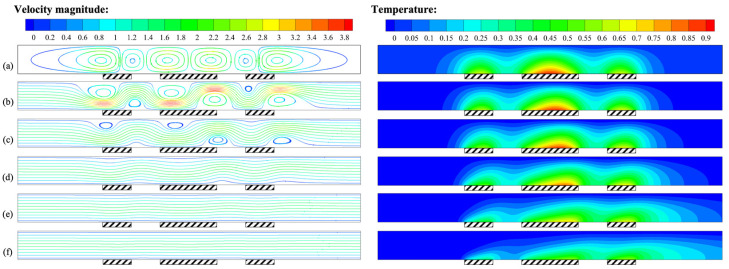
Velocity field and temperature field for *H* = 1 mm, *γ* = 0°, and *P* = 100 mW: (**a**) *Q* = 0 μg/s; (**b**) *Q* = 85.5 μg/s; (**c**) *Q* = 171 μg/s; (**d**) *Q* = 342 μg/s; (**e**) *Q* = 684 μg/s; (**f**) *Q* = 1368 μg/s.

For thermal flowmeters, the mass flow rate is not only the physical quantity to be measured, but also the parameter that changes most frequently during normal operation, and its importance cannot be ignored. Figure 12 shows the flow and temperature fields at different mass flow rates *Q*. When *Q* = 0, the streamlines and temperature distribution in the channel were symmetric, and only buoyancy convection existed. When *Q* was approximately 85.5 μg/s, forced convection began to intervene, the high-temperature region gradually tilted downstream, and buoyancy convection still dominated. When *Q* was approximately 342 μg/s, the streamlines tended to be gentle, and the strength of forced convection was comparable to that of buoyancy convection, representing the critical state of flow transition. As *Q* continued to increase, the streamlines became straight, the isotherms near the heat sources became flat and obviously tilted downstream, and the effect of forced convection became significant. The above analysis indicates that the mass flow rate *Q* was the direct cause of forced convection and had an important influence on its intensity.

**Figure 13:**
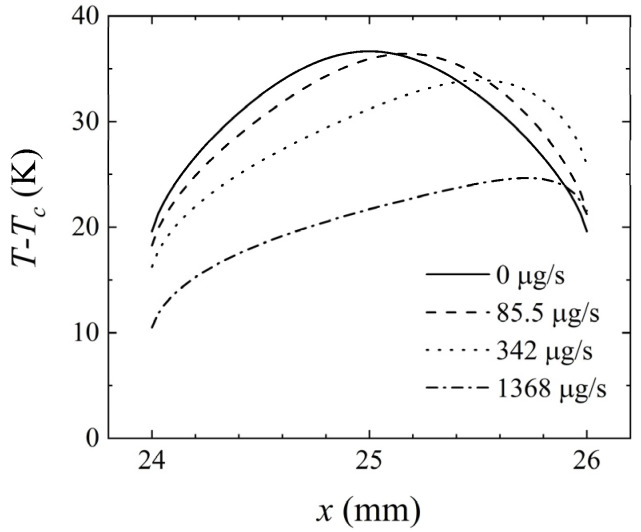
Variation of *T*–*T_c_* at the intermediate heat source (24–26 mm) for *H* = 1 mm, *γ* = 0°, and *P* = 100 mW at different mass flow rates.

At different mass flow rates, the flow pattern varied, leading to differences in temperature at the heat sources. Figure 13 shows the temperature distribution at the intermediate heat source under different mass flow rates. When *Q* = 0 μg/s, the temperature distribution was symmetric, and the fluid was only affected by buoyancy convection. As *Q* increased, the highest temperature decreased, and the high-temperature region shifted downstream as the strength of forced convection increased.

**Figure 14:**
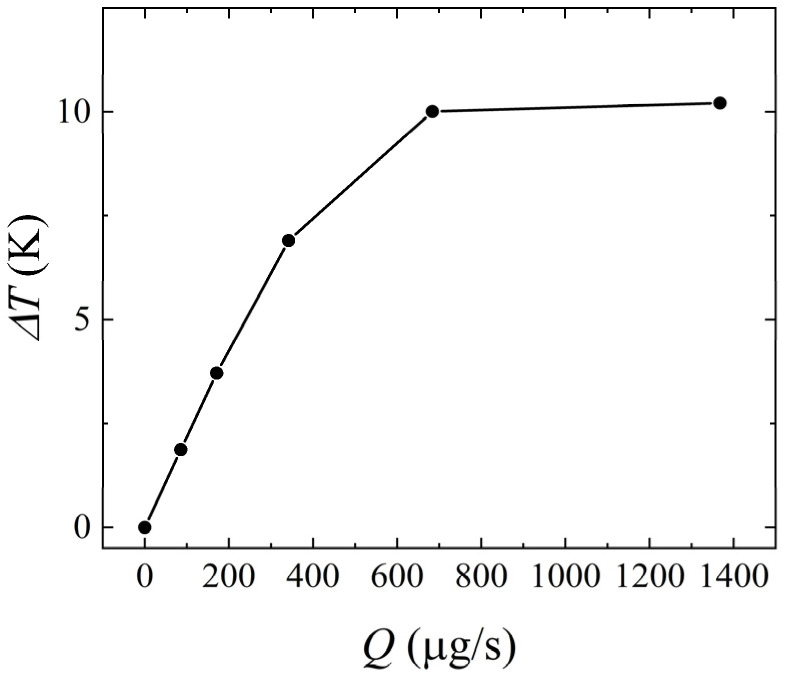
Variation of *ΔT* with the mass flow rate for *H* = 1 mm, *γ* = 0°, and *P* = 100 mW.

Similarly, in order to investigate the variation of the temperature difference between the upstream and downstream heat sources of the thermal flowmeter with mass flow rate during operation, Figure 14 was obtained. When *Q* was less than 342 μg/s, the temperature difference between upstream and downstream heat sources increased rapidly with the mass flow rate, and the measurement sensitivity was higher at this time. When *Q* was greater than 342 μg/s, the rate of increase in temperature difference between the upstream and downstream heat sources slowed down, and the influence of forced convection increased. It is worth mentioning that, in order to ensure measurement sensitivity, the mass flow rate range allowed for the operation of the thermal flowmeter to be the stage with a lower mass flow rate.

The above analysis shows that, as the mass flow rate increased, the forced convection became stronger, leading to a decrease in temperature at the heat sources and an increase in temperature difference between upstream and downstream heat sources.

### 5.5. Heating Power

**Figure 15:**
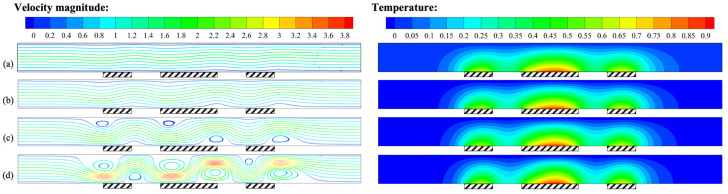
Velocity field and temperature field for *Q* = 85.5 μg/s, *H* = 1 mm, and *γ* = 0°: (**a**) *P* = 12.5 mW; (**b**) *P* = 25 mW; (**c**) *P* = 50 mW; (**d**) *P* = 100 mW.

The previous section investigated the transition of flow patterns by varying the inlet mass flow rate to affect the strength of forced convection. In this section, the influence of different factors on flow pattern transition was studied from another perspective, by changing the heating power of the heat sources to affect the strength of buoyancy convection in the channel. The results of the flow and temperature fields for different heating powers are shown in Figure 15. When *P* < 25 mW, the streamlines were straight, and the buoyancy effect was very weak. When *P* = 25 mW, the streamlines began to bend, and the system was in a critical state of flow transition, with buoyancy convection increasing. When *P* > 25 mW, the flow dominated by buoyancy transformed to steady multicellular flow. Under the influence of buoyancy convection, the upper isotherm of the three heat sources protruded upward.

**Figure 16:**
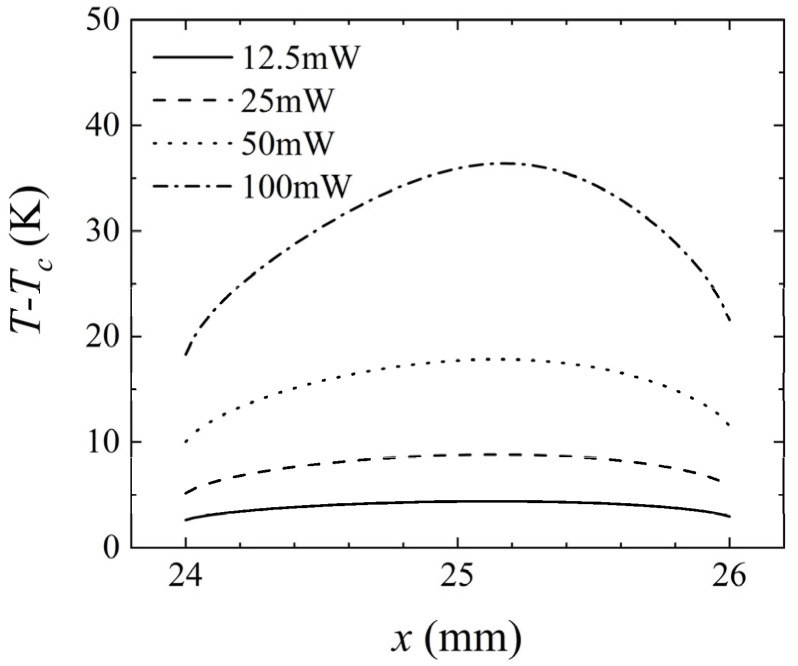
Variation of *T*–*T_c_* at the intermediate heat source (24–26 mm) for *Q* = 85.5 μg/s, *H* = 1 mm, and *γ* = 0°, at different heating powers.

**Figure 17:**
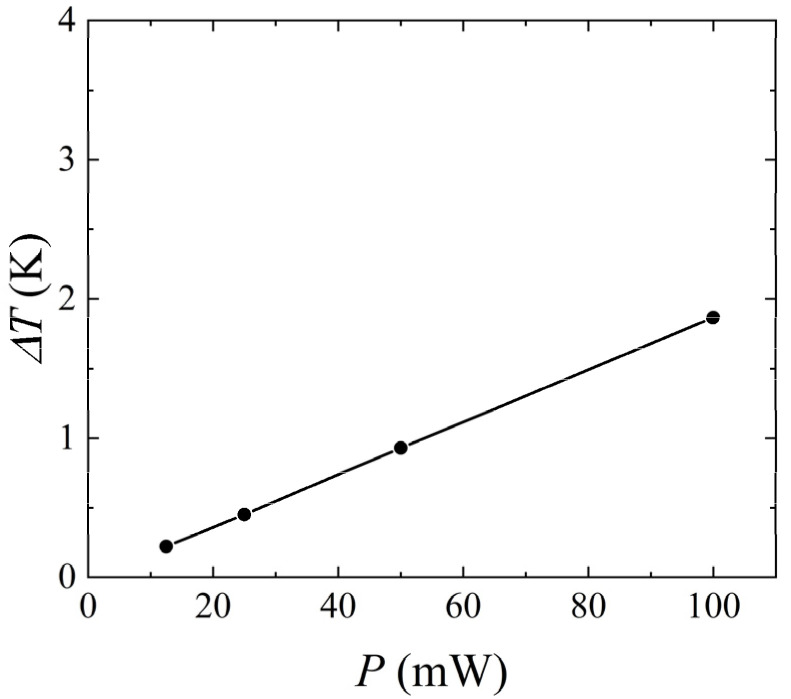
Variation of *ΔT* with the heating power for *Q* = 85.5 μg/s, *H* = 1 mm, and *γ* = 0°.

In order to observe the temperature distribution of the heat sources under different heating powers, Figure 16 was plotted. As the heating power *P* increased, the temperature of the heat source increased. Figure 17 shows the variation of the temperature difference between upstream and downstream heat sources with heating power. As *P* increased from 12.5 mW to 100 mW, the temperature difference between upstream and downstream heat sources increased from 0.22 K to 1.87 K, revealing a linear relationship between heating power and the temperature difference. Therefore, under the premise of ensuring that the measured liquid did not undergo phase change and did not cause a flow pattern transition within the range of mass flow rate measurement, appropriately increasing the heating power could increase the sensitivity of the thermal flowmeter.

### 5.6. Flow Pattern Transition

In the preceding sections of this paper, we investigated the influence of the inclination angle, channel height, inlet mass flow rate, and heating power on the measurement of thermal flowmeters in the normal gravity. The results showed that a higher channel height, smaller mass flow rate, and larger heating power could lead to a transition of the flow pattern, having an adverse effect on the measurement accuracy of the mass flow rate. In fact, there are many other factors that can affect thermal flowmeters, such as the type of liquid working fluid and the channel depth *d_z_*. To assess the influence of different parameters more accurately and uniformly on the strength of the two convection patterns, and to explore the factors that cause flow pattern transitions from a mechanistic perspective, this study introduced the Reynolds number and the Grashof number to evaluate fluid dynamics and heat transfer. Among the factors studied in this paper, the mass flow rate changed the Reynolds number, influencing the strength of forced convection, while the flow channel height and heating power affected the strength of buoyancy convection by changing the Grashof number. Forced convection and buoyancy convection jointly influenced the flow pattern.

In the present work, two different flow patterns were observed: steady multicellular flow, referred to as SMC, and steady unicellular flow, referred to as SUC. The typical flow pattern of SMC is shown in Figure 12b, while the typical flow pattern of SUC is shown in Figure 12f.

**Figure 18:**
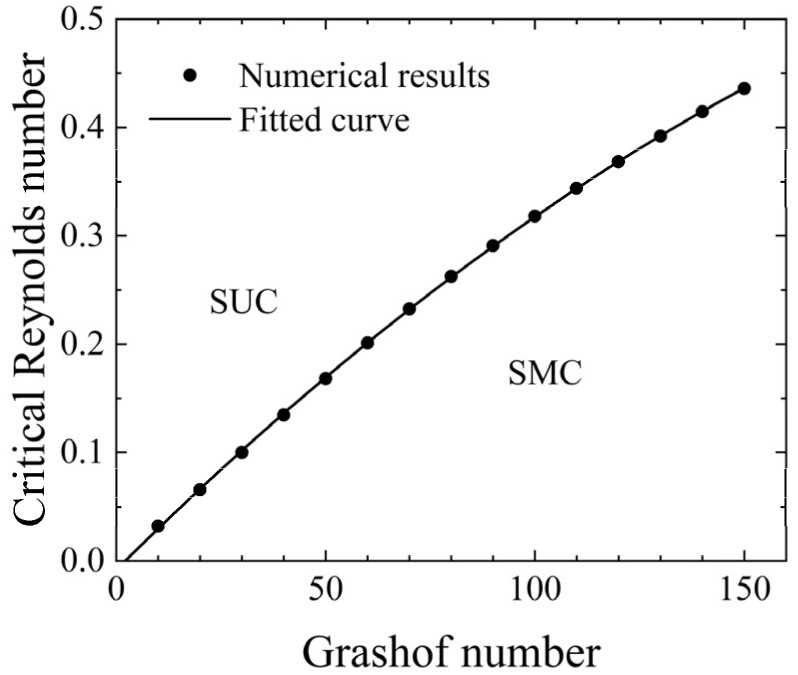
Transition map of flow pattern.

Figure 18 illustrates the relationship between the Grashof number and the Reynolds number at the critical state of flow pattern transition. Through calculation, the Reynolds numbers at which the flow pattern transition occurred were determined for different Grashof numbers. A curve was fitted to represent the relationship between the Grashof number and the Reynolds number at the critical state, and the following relationship was obtained:

(7) $$Re=-5.861\times 10^{-6}Gr^{2}+3.843\times 10^{-3}Gr-8.253\times 10^{-3}.$$

To avoid phase changes, the relationship between the Reynolds number and the Grashof number was fitted using a quadratic polynomial. In this case, the constant term was closer to zero than that of a linear curve, better representing the critical condition where the Grashof number is equal to zero under zero inlet velocity.

As concluded from the previous sections, a transition in the flow pattern could cause changes in the temperature distribution at the heat sources and the temperature difference between upstream and downstream heat sources, resulting in inaccurate mass flow rate measurement. The above equation could be used to calculate the lowest mass flow rate for which the flow pattern remained unchanged when the size and heating power of the thermal flowmeter were determined, i.e., the lower limit of the mass flow rate that could be accurately measured. When the incoming flow’s Reynolds number was small or the Grashof number inside the channel was large, natural convection dominated, and the flow pattern transitioned from SUC to SMC. To achieve higher measurement accuracy and suppress buoyancy convection, it was necessary to increase the mass flow rate, i.e., increase the strength of forced convection.

Among the factors studied in this paper, the Reynolds number was used to describe the changes in mass flow rate, while the Grashof number was used to describe the influence of the heat sources. When designing a thermal flowmeter, all these influencing factors should be considered comprehensively, and the corresponding Reynolds and Grashof values for the range of mass flow rate measurement should be calculated to avoid the transition of the flow pattern as best as possible.

## 6. Conclusions

In summary, this study numerically investigated the factors that influence the measurement of thermal flowmeters, specifically examining the impact of buoyancy convection and forced convection on the sensitivity of flow rate measurements.

The results indicate that gravity level, inclination angle, channel height, mass flow rate, and heating power all impact flow rate measurements by affecting flow patterns and temperature distribution. In the presence of gravity, the convective cells emerge, leading to a greater temperature difference between upstream and downstream heat sources, resulting in an overestimation of the flow rate measurement. As the inclination angle changes from negative to positive, the convective cells move further away from the heat sources. A larger channel height enhances the buoyancy effect, making the flow pattern more susceptible to change, and increasing the temperature difference between upstream and downstream heat sources. At low mass flow rates, the temperature difference has a higher sensitivity to mass flow rate. As the mass flow rate increases, forced convection becomes stronger, resulting in a decreased temperature difference related to lower sensitivity. When the heating power of the flowmeter is high, the buoyancy effect becomes stronger, leading to a larger temperature difference between upstream and downstream heat sources and higher sensitivity of the flow rate measurement.

The present work introduced the evaluation of flow and heat transfer using the dimensionless parameters Reynolds number and Grashof number. The emergence of convective cells affects the accuracy of thermal flowmeter measurement. The critical Reynolds numbers for flow pattern transitions at different Grashof numbers are calculated, and the *Gr*–*Re* relationship is fitted. When the Reynolds number of the channel exceeds the critical value, the flow pattern exhibits steady unicellular flow. On the contrary, the flow pattern is steady multicellular flow, which affects the measurement results.

In this work, the Reynolds number was used to describe the changes in mass flow rate, and the Grashof number was used to describe the influence of the heat sources and the channel height. To ensure the effective design of a thermal flowmeter, it is imperative to consider all these influencing factors comprehensively, and the corresponding Reynolds number and Grashof number for the mass flow rate measurement range should be calculated in order to minimize the transition of the flow pattern.

This paper focused on the two-dimensional numerical simulation of a liquid thermal flowmeter within the scope of low flow rate. To account for more complicated application scenarios, the three-dimensional simulation framework for various working fluids will be considered in future work.

## Figures and Tables

**Table 1:** Physical properties of the working fluid (deionized water). Data from [30].

Fluid	Density *ρ* (kg·m^−3^)	Dynamic Viscosity *μ* (Pa·s)	Thermal Conductivity *k* (W·m^−1^·K^−1^)	Specific Heat *C_p_* (J·kg^−1^·K^−1^)	Coefficient of Thermal Expansion *β* (K^−1^)
Water	997	8.55 × 10^−4^	0.613	4179	276.1 × 10^−6^

**Table 2:** Grid convergence for dimensionless temperature at heat sources.

Grids—*N_x_* × *N_y_*	*θ_L_*	*θ_M_*	*θ_R_*	*θ_max_*
320 × 20	0.3624	0.5894	0.6186	0.6960
390 × 30	0.3623	0.5892	0.6185	0.6960
460 × 40	0.3622	0.5891	0.6184	0.6959
530 × 50	0.3621	0.5890	0.6184	0.6958

## Data Availability

Not available.

## Nomenclature

*l*	Length of the channel (mm)
*H*	Height of the channel (mm)
*H*_0_	Reference height (mm)
*d_z_*	Depth of the channel (mm)
*d*	Length between heat source (mm)
*Δx*	Length of the minimum heat source (mm)
*P*	Heating power (mW)
*q*	Heat flux (W·m^−2^)
*u*_0_	Inlet velocity (m/s)
*t*	Time (s)
***g***	Acceleration due to gravity (m·s^−2^)
***u***	Velocity vector (m/s)
*x*	Streamwise coordinate (mm)
*N_x_*	Numbers of grids in *x*-direction
*y*	Transverse coordinate (mm)
*N_y_*	Numbers of grids in *y*-direction
*p*	Pressure (Pa)
*T*	Temperature (K)
*T_c_*	Ambient temperature (K)
*ΔT*	Temperature difference between upstream and downstream heat sources (K)
*Q*	Mass flow rate (μg/s)
*a*	Thermal diffusivity (m^2^/s)
*k*	Thermal conductivity (W·m^−1^·K^−1^)
*C_p_*	Heat capacity at constant pressure (J·kg^−1^·K^−1^)
**Greek symbols**	
*ν*	*ν = μ*/*ρ,* kinematic viscosity (m^2^/s)
*β*	Volumetric expansion coefficient (K^−1^)
*γ*	Inclination angle of the channel (°)
*ρ*	Density (kg·m^−3^)
*ρ*_0_	Value of the density when *T* = *T_c_* (kg·m^−3^)
*μ*	Dynamic viscosity coefficient (Pa·s)
**Dimensionless values**	
$\bar{l}$	Dimensionless length of the channel
$\bar{H}$	Dimensionless height of the channel
$\bar{u}$	Dimensionless velocity vector
$\bar{p}$	Dimensionless pressure
$\bar{t}$	Dimensionless time
*θ*	Dimensionless temperature
**Dimensionless number**	
*Pr*	Prandtl number *Pr* = *ν*/*a*
*Re*	Reynolds number *Re = Q*/(*μd_z_)*
*Gr*	Grashof number *Gr = gβqρ*_0_^2^*H*^4^/(*μ*^2^*k*)
**Subscript**	
*L*	Upstream source
*M*	Intermediate source
*R*	Downstream source
*max*	Maximum value
